# Physiotherapists’ perceptions of challenges facing evidence-based practice and the importance of environmental empowerment in fall prevention in the municipality – a qualitative study

**DOI:** 10.1186/s12877-020-01846-8

**Published:** 2020-10-29

**Authors:** Hilde Worum, Daniela Lillekroken, Kirsti Skavberg Roaldsen, Birgitte Ahlsen, Astrid Bergland

**Affiliations:** 1 Department of Physiotherapy, Faculty of Health Sciences, Oslo Metropolitan University, Oslo, Norway; 2 Department of Nursing and Health Promotion, Faculty of Health Sciences, Oslo Metropolitan University, Oslo, Norway; 3 Department of Neurobiology, Health Sciences and Society, Karolinska Institute, Stockholm, Sweden; 4 Department of Research, Sunnaas Rehabilitation Hospital, Oslo, Norway

**Keywords:** Barrier, Facilitator, Fall prevention, Implementation, Knowledge translation, Leadership, Organisation

## Abstract

**Background:**

Falls in older adults are an increasingly important public-health concern. Despite abundant research, fall rates have not been reduced, because implementation of evidence-based fall-prevention measures has been slow and limited. This study aims to explore physiotherapists’ perceptions on external factors, such as public policy, organisation and leadership, regarding the relation between knowledge translation and the three elements of evidence-based practice (EBP) to effectively address barriers and facilitate the uptake of EBP in fall prevention.

**Methods:**

We conducted semi-structured interviews with 18 *physiotherapists* (men = 7; women = 11) *working with fall prevention in the primary healthcare system.* The physiotherapists ranged in age from 27 to 60 years (median 36 years) and had worked as a physiotherapist from 1 to 36 years (median 7 years). Data are analysed using thematic analysis.

**Results:**

The analysis revealed one main theme and four sub-themes. The main theme was ‘Environmental empowerment enhances physiotherapists’ capabilities for using EBP’. A resourceful work environment facilitates EBP, having access to information about research-based knowledge, supportive leadership, enough human resources and opportunities to learn and grow at work. The four sub-themes were as follows: 1) ‘Tension between attributes of research-based knowledge and organisational routines and practices’; 2) ‘Evidence must be informed by policymakers—What works?’; 3) ‘Empowering culture and work environment—A steppingstone to EBP’ and 4) ‘Organisation readiness for EBP, managerial and clinical relations’. Success in environmental empowerment depends on the leader’s role in creating preconditions at the workplace that may lead to important positive personal and organisational outcomes for EBP. Two-way communication and transfer-of-information are also key factors in the development of positive work engagement when using EBP.

**Conclusion:**

The findings of this study outline tension between policy, leadership, organisational facilitators and EBP. Leadership is influenced by policy with ripple effects for the organisation and clinicians. Organisational facilitators form structural empowerment, which is the foundation for creating an EBP environment.

**Trial registration:**

2018/2227/REC south-east C. Registered 19 December 2018, Norwegian Ethics Committee for Medical and Health Research Ethics.

**Supplementary Information:**

The online version contains supplementary material available at 10.1186/s12877-020-01846-8.

## Background

Worldwide, governments are exploring innovative and evidence- based ways to improve public services [1]. Evidence-based healthcare has been described as ‘a discipline centered upon evidence-based decision-making about groups of patients, or populations, which may be manifest as evidence-based policy-making, purchasing or management’ [2]. Evidence-based practice (EBP) and knowledge translation (KT) are closely related concepts that pertain the identification, utilisation and application of research-based knowledge in clinical practice. EBP refers to the integration of the best available research evidence with clinical expertise and patient characteristics and preferences [3]. KT emphasises the synthesis, dissemination, exchange and application of knowledge from research findings and other sources to influence changes in practice and improve health outcomes [4]. Grimshaw et al. [5] defined knowledge translation as, ‘ensuring that stakeholders are aware of and use research evidence to inform their health and healthcare decision making’.

Falls in older adults are an increasingly important personal consequences and important implications for health care, both due to the rising number of older adults and the severe consequences, which contribute to the global burden of injuries [6–8]. A fall is defined as “an unexpected event in which the participant comes to rest on the ground, floor, or a lower level” [9]. Several systematic reviews and meta-analyses have provided evidence for the effectiveness of fall-prevention programmes [10]. Moreover, falls are among the most frequent causes of unintentional injuries, such as hip fractures, and falling accounts for half of all hospital admissions of older adults [6]. Approximately 30% of home-dwelling older adults over 65 experience at least one fall yearly, and this increases to 50% for those over 80 [10]. The financial consequences for falls and associated injuries are increasing, and it has been estimated to be 1.5% of health care costs in European countries [11].

KT and the implementation is a multifactorial complex process [12–14]. Although there is ample research, falls have not been reduced, due to the limited implementation of evidence-based fall prevention into practice [15–18]. Several studies have examined the effects of organizational strategies for implementing improvements in patient care and organizational culture and found that factors such as organization and research admissions are of paramount importance to the success of KT and EBP [19–21].

The need to better understand the impact of contextual factors has been emphasised in several reports that described the links between the organisational context and KT, as well as that of EBP [21–25]. Organizational context is understood as the healthcare setting in which EBP is to take place and is related to culture, capacity and infrastructure [23]. Lately, it has been an increasing interest among researchers to focus more on the organisational level and work environment as they relate to enabling the use of EBP [21, 26, 27]. Theories and concepts concerning organisational culture, organisational climate, leadership and organisational learning are relevant to understand and explain organisational influences on implementation processes thereof [12, 21, 27–29]. However, although there are an increasing number of theories, access to information seems to be the most important empowerment method for healthcare practitioners, as well as for empowering work environments. According to Tebbitt [30], ‘empowerment means creating and sustaining a work environment that speaks to values that facilitate the employees’ choice to invest in and own personal actions and behaviours resulting in positive contributions to the organization’s mission’ (p. 19). With regard to an organization mandate, Tebbitt [30] suggested that the empowerment of staff is an important factor, particularly in the face of organizational change.

According to Kanter [31], work environments have the potential to overcome barriers and empower those who need access to information, support and resources that are necessary to accomplish high-quality work, as well as provide opportunities for growth and development of knowledge and skills. Empowering work environments are important because EBP is a problem-solving approach to clinical decision-making in healthcare that integrates the best evidence from well-designed studies with a clinician’s expertise, including internal evidence from patient assessments, practice data and a patient’s values and *preferences* [32].

Leadership influences the work environment and the way employees perceive their work. Leaders with a vision for implementing EBP in the organization can influence the implementation of evidence-based practice by providing sufficient resources, support and integrating evidence into their own leadership practice [32]. Leadership style, type of power and the leader’s commitment are important for knowledge development and can contribute to an organizational climate that facilitates engagement in EBP [21] and improves employees’ internal motivations [33]. Further, employees can experience increased attachment to one’s work by the leader showing his or her attention, set clear expectations, praise employees for good performance and are fair [34, 35].

In a previous study, Worum et al. [36] explored the reflections and experiences of older community-dwelling patients who had experienced a fall regarding EBP in a fall-prevention training programme. Older patients perceived EBP as a seal of approval and a ‘guarantee of high quality’ treatment [36]. Physiotherapists also play an important role in implementing fall-prevention programmes [37]. In a recent study, Cerderbom et al. [38] explored physiotherapists’ views of how they experienced and perceived their role when working with fall prevention in the primary care setting. Their findings disclosed a multifaceted and innovative role that requires competent, experienced and skilful physiotherapists who can cope with the interaction between the micro- (i.e., individual), meso- (i.e., organisational) and macro-levels (i.e., policy). They described how their multifaceted, evidence-based and innovative role means initiating and motivating, both on the individual level (i.e., with an older person) and with their team of colleagues and at the level of the organisation (i.e., policy, work culture). Last but not least, physiotherapists’ success in implementing fall-prevention programmes and interventions depend on empowering leadership and a working culture, along with time and multifaceted professional competence [38]. Furthermore, research indicate that clinicians’ experience might contribute to strategic priorities to improve patient experience and place patients at the centre of healthcare and decision-making [39].

To the best of our knowledge, no study has investigated physiotherapists’ perceptions of factors related KT with respect to organisation, leadership or work environment when focusing on the three elements of EBP—research-based knowledge, professional knowledge and patients’ values and preferences—as they relate to fall prevention in the community. Thus, this study aims to explore physiotherapists’ perceptions on external factors, such as public policy, organisation and leadership, regarding the relation between KT and the three elements in EBP to effectively address barriers and facilitate the uptake of EBP. Using the clinicians’ own perspectives may help us to identify factors that may have a positive impact on the use of EBP. The findings from this study will contribute to gaining knowledge about how to successfully implement EBP, and in doing so, this study will support the development of evidence-based health services available to those who have fallen, health professionals and policy-makers in municipalities.

## Methods

### Design

The study was designed using a qualitative phenomenological approach. The materiel consists of 18 in-depth interviews with physiotherapists in four different districts in Oslo, Norway. A qualitative phenomenological approach offers an opportunity for in-depth information about a phenomenon about which little may be known [40]. A phenomenological perspective explores how human beings perceive the world and transform those experiences into consciousness [41].

As such, a phenomenological approach was considered useful to explore physiotherapists’ perceptions on external factors regarding the relation between KT and EBP with the aim of effectively addressing barriers and facilitate the uptake of EBP. We consider this to be the most appropriate approach, since the aim of the study is to develop descriptions of the phenomenon being studied: physiotherapists’ perceptions on external factors, such as public policy, organisation and leadership, regarding the relation between KT and the three elements in EBP**—**research-based knowledge, professional knowledge and patients’ needs and values—in fall prevention in the municipality.

### Recruitment and participants

Participants were recruited by the first author of this paper through leaders and contacts who attended an informational meeting regarding this study (e.g. aim, reasons for doing the research, personal goals). Subsequently, a response was given to the first author about potential participants who were contacted by either email or telephone. Furthermore, the researchers had no relationship with the participants. We used a purposeful sample to achieve a heterogeneity within the physiotherapist groups, which represented a broad spectrum of health professionals with different experiences and from different contexts to strengthen the validity of the study [40]. We sought a maximum variation in the sample in terms of socio-economic status of the district in which they were employed, gender, age and years of experience. Participants were included if they met two criteria: (i) they had knowledge and experience in practicing fall-prevention interventions, and (ii) they were employed at the municipality health care service. Based on the recommendation from Creswell and Poth [42] regarding inclusion of participants until the researcher assesses the number of informants as a sufficient sample (15 ± 10), data collection continued until the researchers were satisfied that rich detail of the participants’ experiences had been captured. Recruitment was considered complete when saturation was achieved. Data saturation means that no new information or themes are discovered in the empirical data, which indicates that the data collection may finish. At this point, the researcher can be reasonably assured that further data collection will not enhance or change the findings of a study [43].. Furthermore, we used the concept “information power” to guide adequate sample size for qualitative studies. Information power indicates that the more information the sample holds, relevant to the actual study, the lower amount of participants is needed and depends on 1) the aim of the study, 2) sample specificity, 3) use of established theory, 4) quality of dialogue, and 5) analysis strategy [44]. All the participants delivered detailed descriptions, and data were deemed to be rich enough to elaborate a general structure. In total, 18 physiotherapists, seven men and eleven women, from four different districts from the Oslo municipality participated in the study. No participants withdrew from the study. The physiotherapists ranged in age from 27 to 60 years (median 36 years) and had worked as a physiotherapist from 1 to 36 years (median 7 years). Table 1 presents information about the participants’ age, gender, years of experience as physiotherapists, years of experience in the primary healthcare service, additional education and position and/or unit.

**Table 1:** Information about participants

Physio-therapist ID	Gender	Year of graduation	Years of experience	Years working in primary healthcare	Additional education
1	F	1989	30	1	Psychology, 1 year
2	F	1988	27	22	Advanced course in Physiotherapy for Older People Master’s degree in Rehabilitation and Habilitation
3	M	2018	1.5	0.5	–
4	M	2016	3	2	–
5	M	2016	3	2.5	–
6	M	2012	7	7	–
7	F	2012	7	7	–
8	F	2008	7	7	Master’s degree in Public Health
9	F	1998	21	19	Advanced course in Physiotherapy for Older People
10	F	1983	36	30	Psychology (1 year) *Preparatory* test in philosophy Manual Therapy, Part 1 Various courses: cancer, exercise, public health, cardiac arrest, lung disease, dementia, psychiatry
11	F	2015	3.5	3	Elective Orthopaedics for Chronic Disorders Pain Management, Part 1 ‘Active against cancer’ ‘Active with osteoarthritis’
12	M	2007	11	8	–
13	F	2007	4	2	–
14	F	2017	1.5	1	–
15	M	2018	1	1	–
16	F	2010	10	9	–
17	F	1994	25	13	Advanced course in Physiotherapy for Older People (Currently a student in Master’s degree in physiotherapy)
18	M	2018	1.5	1.5	–

### Data collection

The first author (HW) conducted all the interviews. Prior to the interviews, the participants received a thematic interview guide; this was a conscious decision of the research team, as the more-or-less prepared answers ensured completeness. The interview guide consisted of a series of open-ended questions created for this study by the researchers (See Additional file 1). The interview guide was tested in a pilot interview for comprehensibility. Furthermore, prompts were used in all interviews to ensure that all participants were prompted in a similar manner. Prior to the interview, participants completed a paper questionnaire with general information about themselves as physiotherapists, such as years of education, additional education, workplace, etc. Participants were asked to describe current practices and their experiences of EBP in fall prevention, their understanding of how to work EBP, success factors for implementing EBP, the necessary requirements for practising EBP, and the balance between the various elements of EBP. To facilitate a discussion, the illustration of the EBP model devised by Haynes et al. [45] was used during the interviews. The interviews were conducted between May and November 2019 and took place at the physiotherapist’s workplace. All interviews were conducted face to face by the first author and participant. All participants were interviewed only once. The interviews lasted about 45–60 min and took place at the physiotherapist’s workplace. To support the participants’ narratives, audiotape and field notes were used during the interviews. There was no modification of the procedures in response to evolving study findings.

The participants’ perceptions of the interviewer, including her professional role, can influence the interaction, and hence the information that is revealed [46]. In this study, the participants were informed about the researcher’s professional background as a physiotherapist and PhD student. This may have been important in the sense that the informants fail to give detailed descriptions, because they assume that someone as a physiotherapist already has some knowledge of the field of practice.. To avoid this, the participants were asked to describe in detail all the descriptions of clinical practice and any challenges with EBP. On the other hand, the researcher’s professional background and the fact that there was no close relationship between her and the participants may have contributed to they being able to tell something they would not otherwise tell. Challenges experience with EBP practice are of importance for the interdisciplinary team so, hopefully, our results are transferable to other healthcare professional practices. However, if the interviewer has been a nurse or a medical doctor they will have asked other follow up questions and received other answers.

### Analysis

The interviews were audio recorded and then transcribed verbatim by an independent person. Since the transcripts were not sent to the participants, the first author who performed the interviews reviewed the transcripts for accuracy. A qualitative software programme, HyperRESEARCH™, was used to organise data. The first author was primarily responsible for the analysis of the data, but all authors were involved and contributed to the analysis. Thematic analysis with an inductive approach was conducted to analyse the data [47]. During the analysis, data were systematically examined to identify repeated patterns of meaning. This process was composed of Braun and Clarke [47] six-step method: (1) familiarizing the self with the data, (2) generating initial codes, (3) searching for themes, (4) reviewing the identified themes, (5) defining and naming the themes, and (6) preparing the report. Coding at the latent level produced themes that cohered tightly around environmental empowerment and revealed four sub-themes and one main theme that influence implementation of EBP. An example of the process of transforming a quotation into initial code, sub-theme and main theme is shown in Table 2.

**Table 2:** Examples of coding strategy

Quotation	Initial code	Sub-theme	Main theme
*[T] here is a pretty big gap further up in the system of the head of department […] they may not see what we do or the utility of what we do, and there are things that we feel are very obvious and are very important, but I feel that they are not up to that mindset […] so yes, to the top management, I feel there is a big gap.*	Gap between management and clinicians	Organisation readiness for EBP, managerial and clinical relations	Environmental empowerment enhances physiotherapists’ capabilities for using EBP
*[T] he primary education we have as a health professional is very superficial and you do not learn enough about how to acquire research-based knowledge as a clinician.*	Insufficient basic education	Tension between attributes of research-based knowledge and organisational routines and practices	
*[W] hat bothers me the most is that those who really need to understand the research, such as politicians and management, don’t quite see it [and] fall prevention is not as high a priority when it is not a spontaneous economic gain.*	Understanding from politicians	Evidence must be informed by policymakers—What works and what is the cost of the benefit?	
*[Y] ou may have a strong desire to be updated, but you do not have time in a busy day. I spend a lot of time to keep me updated, but I do it at home and people are not expected to spend time in the evenings and weekends, to sit and read or go to classes and pay from their own pockets.*	Time and professional development	Empowering culture and work environment—A steppingstone to EBP	

The participants did not provide feedback on the findings. Therefore, to ensure trustworthiness, the categorisation was discussed within the research team until consensus was reached. Furthermore, the methods and results (including quotations) were described in detail, according to recommendations for consolidating criteria to report qualitative interviews (i.e., COREQ) [48]. All the authors are females, professionally trained educators and health professionals who have a background in physiotherapy and nursing. At the time of the study, all researchers were employed at Oslo Metropolitan University. Except for the first author who is a PhD student, all researchers have long experience with qualitative research.

### Ethical considerations

The study was approved by the Norwegian Ethics Committee for medical and health research ethics (2018/2227/REC south-east C). Verbal information about the study was provided to the participants, and oral and written informed consents were obtained from all participants before data collection. The participants were guaranteed confidentiality and reassured that their participation was voluntary and that they could withdraw from the study at any time without needing to state their reasons. All information was treated confidentially, and personal information was anonymized. Unidentified data is personal information where the name, birth number and other directly personal identifiers have been removed and replaced by a link key / lists of codes, so that the information cannot be immediately linked to the participant. Connection keys / lists of codes were stored separately from the personal data. Anonymized data are information that cannot be used to identify individuals in the data material, neither directly through names, personal ID numbers or other unique characteristics or indirectly via background variables, nor through a list of names / connection key or encryption formula or code. Furthermore, data material was stored on a password-protected data to prevent unauthorized access to information.

## Results

**Table:** 

Sub-theme	Main theme
1. Tension between attributes of research-based knowledge and organisational routines and practices	Environmental empowerment enhances physiotherapists’ capabilities to use EBP
2. Evidence must be informed by policymakers—What works?	
3. Empowering culture and work environment—A stepping-stone to EBP	
4. Organisation readiness for EBP, managerial and clinical relations	

The analysis of the data yielded one main theme and four sub-themes. The main theme addresses the benefits of the *Environmental empowerment on physiotherapists’ capabilities to use EBP*. This theme reveals that a resourceful work environment that has access to information, support, enough human resources and opportunities to learn and grow at work are facilitating EBP.

Our findings indicated *Tension between attributes of research-based knowledge and organizational routines and practices*. Ease-of-use, relevancy and applicability in practice are understood as the readability of research articles, formulations and style of the recommendations, including their complexity. In addition, the scientific validity of research and the relevance and applicability in practice therefore need further critical reflection and time to understand. Lack of time and competence to seek out relevant studies, as well as divergent research results were considered an obstacle to EBP. Moreover, the physiotherapists stated that policy provides preconditions for clinical practice. *Evidence must be informed by policymakers—What works?* To succeed with EBP, it requires that politicians both have evidence-based knowledge and understand the value of fall prevention work. A description of an *Empowering culture and work environment—A stepping-stone to EBP* was expressed as crucial for successful KT. Leaders play an important role in creating empowering workplace preconditions that may lead to positive personal and organizational outcomes of importance for EBP. A strong professional environment and a culture of continuous quality improvement influenced the approach for EBP implementation. Crucial to increase EBP engagement is understanding the relationship between leadership and structural empowerment. *Organizational readiness for EBP, managerial and clinical relations* seemed to be intertwined. Management was identified as a key factor in establishing a change in organisational culture. Promoting organisational innovation, resources and a risk-taking climate builds the organisation’s absorptive capacity. Two-way communication and transfer-of-information are also key factors, as good and open communication strategies were reported to play a critical role in the development of positive work engagement when using EBP. Selected quotes from the interviews are shown in italics with participant identifiers below.

### Tension between attributes of research**-**based knowledge and organisational routines and practices

Features of research-based knowledge were reported to represent difficulties because of the attributes related to the research articles. Tension between research-based knowledge and clinical practice were reported to depend on the feature of the research-based knowledge; for example, ease-of-use, relevancy and applicability in practice are understood as the readability of research articles, formulations and style of the recommendations, including their complexity. In addition, the scientific validity of research and the relevance and applicability in practice therefore need further critical reflection and time to understand.

According to all of our participants, it is crucial for EBP implementation that research is presented in an understandable way. Clinical guidelines were said to provide a concrete and precise description of desired performance; by giving detailed advice on what to do in a given situation and to whom in which patient group and so on helps healthcare professionals determine which factors or conditions should be taken into account. In contrast, several participants described a tension between research-based knowledge and the usability and intelligibility of the research described in publications, such as scientific articles. They expressed that the layout of research articles was too detailed and not relevant to them as clinicians. Some of the research was described as unattractive, ‘untidy’ and ‘twisty’. Comprehensive figures, tables and abbreviations make some articles almost impossible to read and understand. Some of the participants also expressed that professional terminology and language can hinder and reduce their understanding of the research, especially when the professional terminology and literature are written in English. Research written in their native, mother-tongue language was preferred over English literature. One participant expressed frustration about the presentation of the research articles as follows:

*I don’t know how many times I [have] started to read a lot of research papers and not finished them because of [the] complexity of the language they use*. (Physiotherapist No. 5)

A majority of the participants preferred concrete, simple and clear recommendations. The content of research articles was considered by the participants to be of various levels of relevance. Advanced and detailed descriptions of the method were considered less relevant to clinical practice and appeared to be a challenge for the clinicians:

*I don’t know in how many research articles I had to jump right to the results and just gave up because of the way it was written [ … ] It might just be natural that one always skips the method section and those kinds of things, which may not have the same great relevance, but I may read myself a little wild sometimes when intervention is also presented in an article. The way intervention is presented can be very, very unclear and maybe a little incomprehensible, and that’s the essence of what I want to get out of an article, as well as the results, of course. If the intervention part is presented in a cumbersome and bad way, then I really get nothing out of it.* (Physiotherapist No. 5)

Most of the physiotherapists expressed satisfaction with the transferability of research in fall prevention and experienced limited tension between research-based knowledge, professional knowledge and patient needs and values. Their recommendations were based on evidence representing relevant aspects of daily practice. Nevertheless, some mentioned that there are some limitations in the methodology used in research. Research was regarded as a small part of a larger whole and not directly transferable to clinical practice in the municipality. In addition, they expressed that clinical trials are often performed in ‘ideal settings’ with various exercise equipment that is not available for home-based community dwellings.

*[T] he research is done in ideal environments and circumstances where you have the equipment you need [ … ] [T] here are some practical things that make it difficult to transfer it directly [ … ] I definitely feel that there is a gap there between research and practice.* (Physiotherapist No. 3)

Several of the participants also expressed concern about having access to research, which they considered to be a challenge in a busy workday. Comprehensive database search strategies are given lower priority than clinical practice and the encounter with the patient; to overcome this barrier, research must be as accessible as possible and almost ‘served’ to them. The large amount of research and lack of time to search for relevant literature, as well as diverse research results, can also confuse the clinician as to which studies they can rely on. In addition to the need for evidence, EBP requires that professionals are trained and endowed with the necessary skills to search after and critically use evidence. In addition to all of this, some of the research was perceived as vague and not specific enough to meet challenges in clinical practice, as one of the physiotherapists stated:

*[I] t is a challenge that there is so much research, you don’t know if what you’re reading is the answer to everything, or the answer to one thing. There may be many other articles that say something different, so I think that can be a challenge.* (Physiotherapist No. 9)

Research and clinical recommendations demand that the working environment facilitates the acquisition of new competence (i.e., knowledge, skills). However, research and clinical recommendations and can only be followed when the physiotherapist has specific knowledge and skills. One physiotherapist acknowledged the challenges regarding competence and use of research:

*[T] he basic education we have as a health professional is very superficial and you do not learn enough about how to acquire research-based knowledge as a clinician.* (Physiotherapist No. 11)

Tension between research-based knowledge and the individual patient was reported by several participants, who stated the importance of prioritising the individual patient. Some of the participants stated that evidence-based practitioners adopt a process of lifelong learning that involves possibilities for continually posing specific questions of direct clinical importance to clients, searching objectively and efficiently for the best current evidence related to each question and taking appropriate action guided by evidence:

*Sometimes it’s really hard to use research—the patient doesn’t fit in a [mould.] I think the starting point is to try to understand the individual [ … ] The knowledge I have is pretty much general and you can quickly start something that is not feasible in accordance with the patient. I think that’s where the professional judgement appears [ … ] Everything is interwoven, general knowledge of strength and balance will always be present, but after all, it has to be adapted to the individual [ … ] So, practice means to move constantly between different types of knowledge.* (Physiotherapist No. 2)

### Evidence must be informed by policymakers—what works?

A majority of the participants stated that evidence can certainly inform policy, but sound policy requires understanding, values and deliberation with respect to practice. They also suggested that policy can be built on other sources of enlightenment, such as clinicians’ experiences. However, the physiotherapists acknowledged the importance of ‘evidence-based policy’ and associated it with the discourse of ‘what works’. It is important to consider the ways in which institutional mechanisms and government structures translate findings into policy, or not. Financial considerations and lack of personal contact with politicians that leads to mistrust of politicians are some of the factors that were highlighted as being important for evidence-based policy and further EBP.

A tension between therapists using EBP and the management conditions given by persons representing policy was evident in our participants’ statements. Policy provides preconditions for clinical practice; the participants emphasised the impact of power and budget struggles for implementing of EBP. Money is power, and power provides opportunities for influencing fall prevention interventions and programmes. Contextual influences on the roles and processes that support EBP were apparent at the policy level. According to our participants, the influence of formal regulatory schemes seemed to be important for the managers’ decisions regarding EBP implementation. However, politicians and clinicians’ understanding of fall prevention and physiotherapy seemed to be viewed as a ‘culture clash’. One physiotherapist shared her frustration over the politicians and leaders’ lack of understanding and insight into physiotherapists’ competence:

*I have tried to say that strength and balance training are what research shows are the most effective intervention to prevent falls and fractures [ … ] [A] nyway, we are told that we should go out and sprinkle [ … ] So, we need to have bags with sand in the cars and we distribute crampons and things like that and that is great, but most of the accidents happen at home.* (Physiotherapist No. 17)

All physiotherapists were aware of the importance of early intervention to prevent falls and the negative development of physical impairment in the elderly. In addition, long-term prevention of falls is economically profitable. However, according to our participants’ perceptions, this knowledge did not appear to be widespread among politicians. Interventions without visible financial gain were therefore gradually abolished.

*Previously, fall prevention groups have started with a quite high intensity training exercises, but you do not see the economic gain after one year and then they are not prioritized which means that they eventually end.* (Physiotherapist No. 15)

Furthermore, one of the physiotherapists expressed his frustration and annoyance regarding politicians’ lack of knowledge and understanding of the financial benefits of fall prevention:

*[W] hat bothers me the most is that those who really need to understand the research, such as politicians and management don’t quite see it [ … ] [F] all prevention is not as high a priority when it is not a spontaneous economic gain.* (Physiotherapist No. 15)

Participants expressed a lack of cooperation with management and politicians. However, numbers and finances seemed to be of interest, as one of the participants mentioned:

*[T] hey are very fond of numbers, so when they see that a hip fracture costs [ … ] NOK 350 000 for the district during the first year, they are very interested in preventing hip fractures.* (Physiotherapist No. 6)

### Empowering culture and work environment—a stepping-stone to EBP

Regarding preconditions to strategically implement EBP, the physiotherapist emphasised the role of management in terms of facilitating prerequisites, as well as creating a positive culture towards EBP. Several physiotherapists expressed a philosophy and culture of continuous quality improvement, which clearly influenced the approach for EBP implementation. The professional environment and common meeting point in the form of professional meetings were described as something the physiotherapists need to survive and overcome as a professional. Knowledge-sharing in a stimulating and engaging academic environment was described as a social process. Physiotherapists interact in constructing meaning. Development and changes in knowledge, behaviour and work routines are the sum of what the ‘team members’ learn and reflect in practice. A majority of the participants suggested that during job training, it is important to establish a context that facilitates building the capacity to perform EBP.

A strong professional environment is crucial for EBP implementation and is described as a centre of power where both human and professional resources are alternately affected. The physiotherapists expressed that they are inspired and motivated by each other through professional fellowship and discussions. Academic discussions and collaboration provide professional development, and engaged employees are more creative, productive and willing to go the extra mile. One participant expressed how a motivating collegiate environment stimulates service innovation and professional culture:

*If you have a bunch of physiotherapists who only do what is strictly necessary, I think it may not be as expedient in terms of the professional and what you can accomplish as a team at the department level. But if you have a department that is motivated and wants to do a good job and wants to go the extra mile, which I feel we do here, most of us, then I think that you achieve more.* (Physiotherapist No. 4)

Another participant described the importance of the academic work environment for motivation as follows:

*The work environment is really alpha and omega for keeping up the motivation and for wanting to develop oneself and the health service and that you have colleagues who are supportive and open to suggestions.* (Physiotherapist No. 6)

The physiotherapists emphasised that the composition and diversity of different professional groups may enrich EBP in fall prevention; however, this can also act as a detriment to the physiotherapists’ core competence. Without a professional community and competent leadership, the physiotherapists’ centres of power seemed ‘empty’. One said:

*I miss a professional powerhouse, I don’t think I want an exclusivity, but I think you are a little dependent on someone talking together about professional knowledge, research or experience.* (Physiotherapist No. 2)

The physiotherapists described how the organisation has a common responsibility for professional development. Research and knowledge distributed by mail, professional meetings and collegial guidance were frequently used approaches. Although most physiotherapists expressed motivation and commitment, one participant described a lack of motivation and interest in fall prevention and research:

*[I] t may just be commitment or motivation to do it, because I wasn’t very keen to read research and stuff like that, so that might be my problem, but I know I should.* (Physiotherapist No. 13)

Leadership were reported to be associated with EBP-enhancing environments. Several of the participants talked about appropriate staffing, effective decision-making, meaningful recognition, skilled communication and true collaboration. They suggested that it was important that the leader and politician were approachable and trustworthy, as well as good communicators, open-minded, knowledgeable, visible, transparent and responsive to the needs of the staff. One physiotherapist expressed frustration and shared her feelings about how the leader of another profession lacked understanding and support for physiotherapists:

*[T] here is no leader in the leadership team that says that this is important that the physical therapists do [ … ] [T] he head of the department wants us to continue with groups, but they are nurses [ … ] [T] hey think that the physiotherapists are always busy with these groups, why should they keep doing that? Why can’t they help us?* (Physiotherapist No. 17)

Factors belonging to this category were reported to be lack of facilitation and organisation for professional development, including time, capacity and resources to make changes. The physiotherapists described the importance of research for clinical practice, and most of them also expressed an interest in and motivation for professional development. Nevertheless, the physiotherapists described a continuous negotiation process between professional development and daily activities. The participants described tension between professional development and daily routines, and they highlighted dilemmas between prioritising working tasks and finding time for reading research for their own professional development. Self-study and reading professional literature were described as real-time and given less priority.

*[I] t can be a bit difficult even if it is possible, in practice it can be a little difficult to prioritize it while you are at work because you often feels there are other things you should do, even though you may not feel that there are other things that are more important, but if you have an application for someone waiting for a walker, then the person is waiting for you and then you have to do it.* (Physiotherapist No. 3)

Participants expressed tension between professional development and economic resources. Despite strong motivation to stay academically up-to-date, the everyday working life imposes limitations. Support and facilitation for competence development seemed low. A minority of the participants expressed that courses and other academic activities are not recognised by management, so course fees and other costs are therefore not covered. One participant described how lack of support from the workplace seemed like a barrier to their professional development:

*The course must be 100% relevant for the job and it should not cost too much [ … ] [Y] ou might have to take leave without pay, pay the tuition fees and take free that day [ … ] [T] his feels quite difficult [ … ] First you have to lose money, then you have to pay for the course, suddenly it becomes very expensive.* (Physiotherapist No.12)

Several of the physiotherapists expressed a strong inner motivation for physiotherapy and that they spent their free time on professional development. One participant said:

*[Y] ou may have a strong desire to be updated, but you do not have time in a busy day. I spend a lot of time to keep [ … ] updated, but I do it at home and people are not expected to spend time in the evenings and weekends, to sit and read or go to classes and pay from their own pockets.* (Physiotherapist No. 11)

However, several participants expressed the need for leadership from the management regarding professional development and facilitation. Research updates as a mandatory task was highlighted as a suggestion for getting more current research into use.

### Organisation readiness for EBP, managerial and clinical relations

EBP was reported to require changing existing routines and habits and leaving what is seen as common practice towards the target group. Promoting organisational innovation, resources and a risk-taking climate builds the organisation’s absorptive capacity. The physiotherapists discussed several factors that are important for the changing culture in organisations. Research and changes might provoke negative reactions among colleagues because the updates are not compatible with their views, position or tasks.

The participants also expressed that their organisation’s readiness for evidence-based fall prevention depended on the individual’s interest and motivation for new knowledge and willingness to make changes, as well as the organisation’s resources, such as time and capacity. Although most of the participants acknowledged that a professional academic environment dedicated to development and research promotes EBP, a minority of the physiotherapists emphasised tension between colleagues, which was created by the composition and the positioning of personalities within the team, and which were described as preconditions for changing the culture within an organisation. The following quote is from a participant who described how hierarchy and long-working seniors can act as a barrier when implementing new knowledge:

*I think it has quite a lot to say, what kind of personalities work together in a place [ … ] especially if it’s a bit like a hierarchy [ … ] [I] f you have a person who has worked for many years and has a lot of experience and further education behind them, then it can be a little difficult to try to change that person’s practice [ … ] [Y] ou really get into routines and maybe not everyone is equally open to new things and then it’s a bit scary as a recent graduate [to] question how things are done.* (Physiotherapist No. 14)

Furthermore, management was identified as a key factor in establishing a change in organisational culture. Openness to change and reflexive practice regarding the relationship between research-based knowledge, professional knowledge and the patient’s need were highlighted as being crucial features for the organisation. One participant described tension between EBP and willingness to change, as well as the ability to take chances:

*[F] irst and foremost, you have to have a leader who leads [ … ] [T] he leader has to be open for change, and maybe lead how to present it [ … ] I don’t know how to create a good culture for it, but the employee has to find it fun being able to change the way you work and maybe see that things are done more efficiently. Be willing to throw things up in the air and take a look it again.* (Physiotherapist No. 8)

One participant pondered the gap between research and practice and raised a possible idea for clinicians who do not feel the need for professional development or improvement; in doing so, he also took a critical look at his own professional practice, which seemed to be influenced by tension between EBP, routines and long clinical experience:

*You already have the recipes for most of the issues you encounter, and based on the experience and motivation, you assume what fails the patient and if the patient is motivated, then you start without feeling being good enough to be up to date on what recent research shows [ … ] I have been working here for eight years now, so I’m starting to feel that I need to be a little attentive [ … ] so that the workplace routines will not define me (or my clinical practice).* (Physiotherapist No.12)

During the interviews, several participants suggested that a possible strategy to address good managerial and clinical relations would be to redistribute healthcare resources wherever and whenever possible and to consider task-shifting of roles when appropriate. Strategic long-term planning and budgeting to provide necessary resources should also be assessed. Further, they mentioned that leadership seemed to have several implications for EBP implementation. The participants described leaders’ tasks as providing personal recognition and support and enabling vision, motivation and facilitation, as well as setting the direction of EBP service. In order to achieve successful implementation, research and professional development must be ‘anchored’ in management. Provisions and mandatory tasks from the leadership are perceived as facilitators for research, as one of the participants stated:

*[I] t depends if management actively advocates that this should be part of our way of working, our culture and devoting time and resources to actually being able to become smarter.* (Physiotherapist No. 11)

The participants agreed upon the idea that different leaders, different levels of service and different professions also involve different understandings of physical therapy with respect to more discussion on topics such as research-based knowledge, professional knowledge and the patient’s need. The participants expressed that some leaders lacked insight into the physiotherapists’ clinical practice, and that leaders need to understand their team members in terms of their needs and encourage them in their professional development. Our participants expressed a desire for increased knowledge among the leaders about physiotherapy and physiotherapists’ tasks. One of the participants said:

*[T] here is a pretty big gap further up in the system of the head of department [ … ] [T] hey may not see what we do or the utility of what we do, and there are things that we feel are very obvious and are very important, but I feel that they are not up to that mindset [ … ] [S] o yes, to the top management, I feel there is a big gap.* (Physiotherapist No. 8)

A majority of the participants also mentioned the importance of transparency and open communication between leaders and employees. They referred to extensive two-way communication and transfer-of-information being the key factors for success when performing EBP. One participant expressed the importance of having a leader from the same profession:

*[W] e have a leader who is a physiotherapist, he is very clever [and] he understands what we are talking about when it is related to physiotherapy. He has a lot of opinions and knowledge. I think it is only advantageous for us to have a leader who is a physiotherapist.* (Physiotherapist No. 4)

## Discussion

The aim of this study was to explore physiotherapists’ perceptions on external factors, such as public policy, organisation and leadership regarding the relation between KT and the three elements in EBP—research-based knowledge, professional knowledge and patients’ needs and values—in fall prevention in the municipality. Overall, the findings emphasised possible tension between performing EBP and available resources. Limited environmental resources were considered necessary to enhance physiotherapists’ competence in EBP. Physiotherapists need more knowledge and skills in managing EBP, as well as organisational factors, such as culture, environment, resources, positive expectations and organisational mandates to implement EBP.

Furthermore, in our findings, public policy proved to be important as it relates to the organisation’s possibilities for activities that are relevant for EBP. As such, the findings indicated the complexity of interacting factors at the micro- (i.e., individual), meso- (i.e., organisational) and macro-levels (i.e., policy) to successful implement EBP in fall prevention. Our findings resonate with the statement from Greenhalgh [21], which asserted that policymaking is about defining and pursuing the right course of action in a particular context at a particular time for a particular group of people with a particular allocation of resources. Furthermore, Greenhalgh [21] claimed that the volume of evidence now available is often large and thus difficult to cope with for clinicians who want their practice to be evidence-based. A large number of reports indicated that: (a) inadequate knowledge and skills in EBP by clinicians, (b) lack of EBP mentors and practice facilitators, (c) misconceptions that EBP takes too much time, (d) cultures and environments that do not support EBP, (e) inadequate resources and (f) a lack of expectations and organisational mandates to implement evidence-based care hinder successful EBP implementation [32, 49–51]; these facilitators and barriers were supported by our findings.

Findings from the current study showed close links between politics, leadership and clinical practice. This in line with Cerderbom et al. [38], who stated that competent, experienced and skilful practitioners and physiotherapists in fall prevention had to cope with the interaction between the micro- (i.e., individual), meso- (i.e., organisational) and macro-levels (i.e., policy) in order to evolve their multifaceted, evidence-based and innovative physiotherapy practice. Furthermore, Cerderbom et al. [38] emphasised the importance of having the capability to make changes and improve professional work in accordance with evidence-based knowledge and that successful fall prevention depends on an empowering leadership and working culture. In line with findings from Cerderbom et al. [38], the participants in our study described the ways in which politicians’ decisions influenced their clinical practice. However, as reported in our findings and in the study by Cerderbom et al. [38], a positive policy environment at the macro-level is required to support the meso- and micro-levels of fall prevention; this includes leadership, financial support and human resources allocation.

Our findings showed that politicians’ lack of knowledge about the effect of fall-prevention measures had a negative impact on the physiotherapists’ mandate and motivation to work in an evidence-based manner. To the physiotherapists’ disappointment, they considered the implementation of policy to lack the integration of research and them as professional experts in the field of fall prevention. Thus, the lack of collaboration between physiotherapists, leaders and policymakers resulted in EBP implementation problems in the community. The physiotherapists expressed frustration at the political decisions that inhibited EBP implementation. Despite the great demand and interest among both patients and physiotherapists, our findings showed that interventions without immediate financial gain were gradually abolished (e.g., group exercise interventions). However, group training is both effective and economic, and when the economic savings were communicated to the politicians, it aroused interest. The description of how decisions are made and/or how change is implemented is consistent with a ‘top-down’ approach, which relies on a leader or leadership making the decisions for how something should be done, and as such affects the tasks of lower-level employees. According to Crinson [52], implementation of policy is presumed to be a ‘one shot’ process that involves the implementation in its entirety of a clear-cut entity (i.e., the policy). However, the real-world is different and challenges this approach [52]. Politicians can have different incentives that are both for and against implementation. Additionally, this process requires negotiation between the different players, and a lack of collaboration can lead to implementation problems [52].

In line with our findings, Worum et al. [36] stated that there are tensions between the different sources incorporated in EBP and the features of evidence in clinical practice. One of these tensions was related to the identification and integration of relevant research, which is one of the EBP dimensions, and a busy everyday life. The availability of research was considered to be a challenge in a busy workday. The physiotherapists attributed this to the large amount of research, comprehensive search strategies in databases and lack of expertise to interpret research, as well as diverse research results. Iles and Davidson [53] found that physiotherapists’ main barriers to EBP were lack of access to easily understandable summaries of evidence, limited journal access and a lack of skills in searching and evaluating research evidence. Nevertheless, our participants stated that even if they understood the process of critically appraising a research article, it was still considered a difficult and time-consuming process. Regarding integration of research into their daily routine, the physiotherapists described a continuous negotiation process between professional development and daily activities and other competing demands, such as applications for aid and other incidental administrative tasks. Finding time to update themselves on research was difficult in a busy day. Despite their awareness of the importance of research-based knowledge, their daily routines proved to be the superior winner. Lack-of-time is a common barrier cited worldwide in healthcare [15, 25, 54] and is one of the most frequent barriers to EBP implementation [55]. In addition, the given workload and routine demands in clinical practice were reported to serve as a barrier in several studies [25, 56]. Our findings are supported by findings from other studies that suggested that neither clinicians nor leaders perceive EBP as being a core in the daily work, and it is therefore given a lower priority when weighed against clinical practice [49, 57].

In line with our findings, Newhouse et al. [58] stated that in order to move EBP implementation forward, leadership must ensure that the appropriate infrastructure is available and supported; this organisational infrastructure consists of human and material resources and a receptive culture. In line with Frambach and Schillewaert [59], the physiotherapists revealed the importance of organisational facilitators, support, leadership, organisational culture and climate in an evolving professional environment seeking to build organisation absorptive capacity for EBP. In line with several studies that presented and explained theories and frameworks of EBP implementation in healthcare [60–63], our findings showed that EBP implementation in an organisation was strengthened through social influences in terms of culture, climate and norms. The physiotherapists described knowledge-sharing in a stimulating and engaging academic environment as being a social process. Individual competence is inherently bound to the organisations` competence, and each reciprocally strengthens the other’s competences through interactions and discussions.

Kanter [31] focused on the presence of social structures in the workplace that enable employees to accomplish their work in meaningful ways. The physiotherapists interacted in constructive meaning and throughout the process, thereby influencing their respective organisations to generate more knowledge (e.g., through professional meetings, supervision, dissemination of research) and achieve more, as well as strengthen EBP practices. These findings support the proposals of Brekke et al. [64] and Rogers [65], who noted that interpersonal contacts within organisations are important influences on the adaption of new behaviour. Furthermore, the physiotherapists described how they, as a unit and organisation, shared a common responsibility for professional development, including distributing research and translating research into practice. A key factor for professional development was the presence of a strong professional environment; thus, a multidisciplinary professional environment with a smaller number of physiotherapists was perceived as being fragmented and expressed as being a weakness for the profession.

Realising the ideals of EBP demands an interest in both learning and reading research, as well as a willingness to make changes. Organisational readiness refers to the extent to which an organisation is willing and able to implement and sustain a particular intervention [66], as successful implementation relies on broad-based motivation and support [67]. Individual readiness to change appears to be critical in successful implementation of changes in organisations [68, 69]. Work attitudes, such as job satisfaction, organisational commitment and a further willingness to exert considerable personal effort on behalf of one’s organisation has been noted as a characteristic of individuals wishing to promote EBP [70]. Furthermore, researchers have emphasised that organisations that start with good knowledge and skills can incorporate new knowledge, are highly specialised and have mechanisms in place to spread knowledge throughout the organisation, and as such are much more likely to explore EBP and eventually initiate the strategies [71–73].

Although a strong professional environment seemed to have a positive influence on clinical practice, the individual physiotherapist requires motivation and an interest in professional development. Dannapfel and Nilsen [74] suggested that EBP issues seem to depend on committed individuals (i.e. leaders, physiotherapists) who have a special interest in EBP. They stated that members of an organisation whose commitment is based on genuine interest (i.e., intrinsic motivation) to perform a certain behaviour, rather than a felt need or pressure, are more likely to champion and promote the value of the behaviour to their colleagues [74]. The majority of physiotherapists seemed to have a positive opinion about EBP, which is in line with several studies [55, 75]. In contrast, a recent systematic review found that providers’ beliefs and attitudes have impeded primary care providers from implementing fall prevention practices [25].

Despite a positive attitude and belief in research, however, some of the participants in our study, especially those with lengthy work experience, reported a lack of interest in research and stated that they did not consider the research to be sufficiently interesting. In line with several studies, the physiotherapists argued that the practice in such cases was based on knowledge gained during initial physiotherapy education and/or personal experience, rather than research-based knowledge [38, 76]. In the clinicians’ defence, Mantzoukas [77] argued that practitioners are busy professionals dealing with complex and unique clinical problems that require on-the-spot decisions. It is therefore virtually impossible to stop before every decision is made and retrieve and appraise all relevant randomized controlled trials [77].

A number of studies identified leadership as being a facilitator in healthcare implementation processes [21, 78–80]. The current study showed that leadership is crucial for establishing a changing organisational culture and enhancing the environment for EBP implementation. The physiotherapists’ perceptions of the absence of empowering conditions in the workplace, including management of organisational facilitators, were highlighted as being crucial for successful implementation. According to Kanter’s Theory of Structural Power [31], a leader’s effectiveness and an employee’s job satisfaction is influenced by empowerment that comes from formal and informal systems within an organisation. Formal systems involve power allocated to positions that are central to the organisation, whereby the power is necessary to achieve the goals of the organisation [31]. Informal power develops from and are long-term and stable connections (e.g., leaders and colleagues), who provide approval, support or information, so individuals may meet goals within collaborative work environments that promote success [31].

According to Kanter [31], employees who have access to high degrees of formal and informal power within their organisation have increased access to the workplace empowerment structures of opportunity, resources, information and support. In return, these empowerment structures shape work attitudes or behaviours, and ultimately, work effectiveness. Some of the physiotherapists described the ways in which the informal power of trust-based leadership impacted EBP. They stated that they felt empowered as physiotherapists and experts on fall prevention with a mandate to assure quality and develop interventions in line with EBP; as an example, in the districts where trust-based leadership was valued, group exercise sessions were strengthened according to patients` preferences.

Kanter [31] explained that empowered individuals are more productive in their job responsibilities and in their advancement within the organisation. Having access to resources, information and support provides opportunities for individual growth and development of skills and in turn, job satisfaction; in contrast, when individuals do not have access to these sources, they feel powerless [31]. In this manner, professionals need to be trained in the necessary skills to find and critically use evidence. The participants in the current study suggested a lack of support for professional development. Indeed, one physiotherapist stated the irony of self-financing and use of vacation time with regard to courses and education, which highlights the need for paid in-service time or opportunities for continuing education without loss of income.

Finally, our participants were concerned about actively engaging leadership within the implementation of EBP. The leader’s ability to express personal recognition and support and enabling vision, motivation and facilitation were characteristics that described satisfactory leadership in terms of engagement. The same line of reasoning was offered by Helfrich et al. [81], who stated that leadership commitment and active interest are behaviours that can positively affect the effectiveness of EBP implementation.

Our findings must be seen in the light of how long time it takes for scientific evidence to be implemented in clinical practice. The most frequently quoted statistic on the gap between researchers and practitioners is that it takes 17 years for research evidence to reach clinical practice [82]. Morris et al. [15] aimed to review the literature describing and quantifying time lags in the health research translation process. However, to understand lags has been limited by the weaknesses of existing data. Fortunately, research has since emerged on a number of barriers that inhibit / facilitate the implementation and KT of research into practice. According to our findings, the gap between research and practice is attributed to many proximal factors, including inadequate practitioner training, a poor fit between treatment requirements and existing organizational structures, insufficient administrative support, and practitioner resistance to change. These findings are congruent with previous studies [58, 59, 68, 69]. Whether these barriers alone can be attributed to the 17-year gap is difficult to say, but probably an important contribution to shorten the gap.

### Strengths and limitations

The strengths of this study are as follows. First, the interview participants were from different districts with different socioeconomic statuses; and second, the participants varied in gender, age and years of experience. Thus, we were able to achieve an in-depth understanding of the barriers to EBP implementation from various points of view and thereby increase the transferability of the findings. There were also a few limitations in this study, such as only physiotherapists’ perspectives being included in this study. To gain a better understanding of the underlying process and the tension between the macro-, meso- and micro-levels, it would have been beneficial to include leaders and politicians in this study. We therefore recommend future studies to explore the relationship between these levels in order to bridge the EBP gap.

## Conclusion

Although the majority of healthcare professionals appear quite EBP-minded and the uptake of EBP is progressing as it relates to fall prevention in the community, important barriers are still obstructing full implementation of EBP in daily clinical practice. The findings of this study outline tension between policy, leadership, organisational facilitators and EBP. Leadership is influenced by policy with ripple effects on the organisation and clinicians. Organisational facilitators form structural empowerment, which seems to be the foundation of creating an environment for EBP. In terms of clinical implications, this study suggests a need for increased collaboration between the macro-, meso- and micro-levels, as well as increased research-based knowledge and EBP at all levels within fall prevention interventions in the community.

## Supplementary Information

**Additional file 1.** Interview guide.

## Data Availability

The dataset supporting the conclusions of this article can be obtained by contacting the first author, Hilde Worum.

## Abbreviations

EBP: Evidence-based practice
KT: Knowledge translation
