# Supplementary material for: Physiotherapists’ perceptions of challenges facing evidence-based practice and the importance of environmental empowerment in fall prevention in the municipality – a qualitative study
Source: BMC Geriatr. 2020 Oct 29;20:432. doi: 10.1186/s12877-020-01846-8 (PMC7596977; doi:10.1186/s12877-020-01846-8)
Supplement: Supplementary file 1 — Additional file 1. Interview guide. [file 12877_2020_1846_MOESM1_ESM.docx]

| **Interview guide**   - Can you please describe your experience of participating in the exercise intervention from start to finish?   - Can you please describe as detailed as possible your experiences of the content of the intervention regarding different exercises, for example balance and strength exercises?   - Can you please describe as detailed as possible your experiences with the therapist?   - Can you please describe as detailed as possible how you experienced that the content of the exercise intervention fit with your preferences?   - Can you please describe as detailed as possible the importance of that the exercise intervention is based on research? - Can you please describe your confidence in research on fall prevention?   - Can you please describe your reflections on treatments that are not based on the best evidence of/ in research?   - Can you please describe your reflections on treatments that are based on the best evidence of/ in research? - Can you please describe qualities of a good therapy?   - Can you please describe your thoughts/ reflection on the importance of research-based knowledge?   - Can you please describe your reflection on the importance of the physiotherapist clinical expertise?   - Can you please describe your reflection of the importance of how your own preferences are met/ taken into account? - Can you please describe what motivates you to participate in fall-prevention intervention?   - Can you please describe what is most important to you about the intervention?   - Can you please describe what the intervention means to you? - Is there anything else you would like to share? |
| --- |
|  |
